# Tongxinluo for the Secondary Prevention of Atherosclerotic Disease: A Systematic Review and Meta‐Analysis of Randomized Clinical Trials

**DOI:** 10.1002/brb3.71232

**Published:** 2026-03-12

**Authors:** Yanyan Feng, Zhaobo Shi, Jiewen Zhang, Jiejie Li

**Affiliations:** ^1^ Department of Neurology The People's Hospital of Zhengzhou University & Henan Provincial People's Hospital Zhengzhou China; ^2^ Department of Neurology Kaifeng Central Hospital Kaifeng China; ^3^ Department of Neurology Beijing Tiantan Hospital Capital Medical University Beijing China

**Keywords:** ASCVD, MACE, stroke, tongxinluo

## Abstract

**Background:**

Cardio‐cerebrovascular events continue to pose major life‐threatening risks worldwide. Though approved for treatment of angina pectoris and ischemic stroke, the effects of tongxinluo, as a traditional Chinese medicine, on stroke and major adverse cardiovascular events (MACE) after atherosclerotic cardiovascular disease (ASCVD) remain controversial.

**Objective:**

This study aims to assess the efficacy of tongxinluo in the secondary prevention of ASCVD.

**Methods:**

The present systematic review was conducted following PRISMA guidelines and the *Cochrane Handbook for Systematic Reviews of Interventions*. In this study‐level meta‐analysis, we conducted a comprehensive search of medical libraries and clinical trial registries, spanning from their inception until November 20, 2024, and randomized controlled trials (RCTs) comparing tongxinluo versus no tongxinluo in a secondary prevention. The ASCVD population was included. The primary outcome was stroke and MACE. The secondary outcome was MACE and myocardial infarction.

**Results and conclusion:**

Four RCTs, including 7087 patients with known coronary artery disease, ischemic stroke, or carotid artery plaque, were included in the analysis. Compared to placebo, tongxinluo numerically decreased the risk of stroke with a RR of 0.84 (95% CI: 0.61–1.17, *p* = 0.31). For the outcome of MACE, tongxinluo reduced the risk by 28% (RR: 0.72, 95% CI: 0.59–0.88, *p* = 0.001). Patients randomized to tongxinluo had a RR of 0.70 (95% CI: 0.54–0.92, *p* = 0.01) for cardiovascular mortality and 0.74 (95% CI: 0.56–0.92, *p* = 0.01) for all‐cause mortality. In summary, in patients with ASCVD, tongxinluo tended to decrease the incidence of stroke, and reduced the composite of stroke, myocardial infarction, and cardiovascular mortality by 28%.

## Graphical abstract

This systematic review and meta‐analysis aims to evaluate the efficacy of tongxinluo in the secondary prevention of atherosclerotic cardiovascular disease (ASCVD). In summary, among patients with ASCVD, tongxinluo was associated with a trend toward a reduced incidence of stroke and a 28% lower risk of major adverse cardiovascular events (MACE), which supports its potential role in the clinical management of patients with atherosclerotic disease.

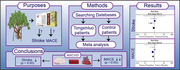

## 1 Introduction

Stroke and coronary disease pose significant life‐threatening conditions globally (Roth et al. 2015). Despite the increasing availability of effective treatment strategies, cardio‐ and cerebrovascular diseases have continued their decades‐long upward trend worldwide, contributing substantially to disability and the global burden of disease (Roth et al. 2020). Therefore, there is an urgent need for additional effective and affordable methods to address the escalating mortality and burden associated with these conditions.

Atherosclerosis stands as a prominent cause of stroke and coronary disease (Frostegård 2013; Herrington et al. 2016). Extensive research has illuminated its nature as a chronic inflammation condition (Björkegren and Lusis 2022; Xing and Lin 2024). At the molecular level, inflammation factors such as interleukin (IL)‐1β and IL‐6 are intricately liked to the initiation, progression, and acute rupture of atherosclerotic plaque (Ridker and Rane 2021). Furthermore, high‐sensitivity C‐reactive protein (hsCRP), serving as a biomarker for systemic inflammation, is increasingly recognized as a pivotal force driving the progression of atherosclerotic cardiovascular disease (ASCVD) and plaque destabilization (Kraaijenhof et al. 2024; Zhang et al. 1999; Mazhar et al. 2024). At the cellular level, a myriad of cells including macrophages, T cells, endothelial cells, and smooth muscle cells are abundant within atherosclerotic plaques in humans, exerting either pro‐atherosclerotic or anti‐atherosclerotic effects during plaque formation and development (Xing and Lin 2024; Saigusa et al. 2020). Tongxinluo, a traditional Chinese medicine, comprises a formulation of 12 ingredients derived from plants and animals, including *Panax ginseng* C.A.Mey. (Ren Shen), *Hirudo nipponica* Whitman (Shui Zhi), *Buthus martensii* Karsch (Quan Xie), *Paeonia lactiflora* Pall. (Chi Shao), *Cryptotympana pustulata* Fabricius (Chan Tui), *Eupolyphaga sinensis* Walker (Tu Bie Chong), *Scolopendra subspinipes mutilans* L. Koch (Wu Gong), *Santalum album* L. (Tan Xiang), *Dalbergia odorifera* T.C.Chen (Jiang Xiang), *Boswellia carterii* Birdw. (Ru Xiang), *Ziziphus jujuba* Mill. (Suan Zao Ren), and *Dryobalanops aromatica* C.F.Gaertn. (Bing Pian), and was approved by the State Food and Drug Administration of China for the treatment of angina pectoris and cerebral infarction for nearly 30 years (Karalliedde and Kappagoda 2009). Studies have shown tongxinluo's efficacy in reducing hsCRP levels, inhibiting plaque progression in ASCVD patients (Zhang et al. 2019; Zhang et al. 2018), and enhancing plaque stability by targeting multiple inflammatory pathways in animal models (Chen et al. 2009; Chen et al. 2021; Zhang et al. 2009; Jiang et al. 2023; Ma et al. 2019; Qi et al. 2022; Zhang et al. 2014). Next, tongxinluo has garnered a series of approvals and recommendations for its application in ASCVD treatment (Karalliedde and Kappagoda 2009; Chen et al. 2009; Dan et al. 2022; Huo et al. 2024).

In the randomized controlled trial (RCT) known as China tongxinluo study for myocardial protection in patients with acute myocardial infarction (CTS‐AMI), the tongxinluo demonstrated a significant reduction in the incident rate of major adverse cardiac and cerebrovascular events (MACCE) and stroke among patients with acute myocardial infarction (Yang et al. 2023). However, in the tongxinluo capsule in ischemic stroke patient (TISS) trial, while the tongxinluo notably improved neural functional outcomes in patients with ischemic stroke, it failed to impact new stroke events or the emergence of new combined clinical vascular events, which encompass ischemic stroke, hemorrhagic stroke, transit ischemic attack, myocardial infarction, and vascular death (Dong et al. 2024). In this study, we conducted a systematic search of RCTs on the use of tongxinluo for the treatment of ASCVD. Through this systematic review and meta‐analysis, we aimed to assess the effectiveness of tongxinluo in preventing stroke and major adverse cardiovascular events (MACE), as well as to distinguish its significance in reducing serious safety events. The primary efficacy outcome was stroke and MACE. The secondary efficacy outcome was defined as 3‐point MACE, defined as stroke, myocardial infarction, or cardiovascular mortality. We used the definitions of outcomes utilized by the original trials. The safety outcomes were death and all‐cause death. Trials for which outcome data were unavailable were excluded from that specific outcome.

## 2 Methods

The protocol for this study has been registered in PROSPERO with the registration number CRD42024621254. Our analysis adhered to the Preferred Reporting Items for Systematic Reviews and Meta‐Analyses (PRISMA) guidelines (Matthew et al. 2021) and the *Cochrane Handbook for Systematic Reviews of Interventions* (Version 6.5, 2024) (Higgins et al. 2024).

### 2.1 Search Strategy

A systematic search of Medline, Web of Science, Embase, the Cochrane Library, Wanfang Med Online, ClinicalTrials.gov, Scopus, and China National Knowledge Infrastructure (CNKI) was conducted from inception to November 20, 2024, to identify RCTs without language restrictions. The key search terms included “tongxinluo,” “randomized clinical trial,” “atherosclerosis,” “stroke,” “cerebral infarction,” “cerebral hemorrhage,” “myocardial infarction,” “peripheral artery disease,” “coronary revascularization,” and their synonyms. These terms were combined using the Boolean operator “AND” (details provided in Supporting Information S1).

### 2.2 Selection Criteria and Data Extraction

In accordance with the expert recommendations on the clinical application of tongxinluo capsules in atherosclerotic cerebrovascular disease (Dan et al. 2022), which recommends a 28‐day course to improve neurological outcomes, RCTs were eligible for inclusion if they compared tongxinluo to a placebo or no tongxinluo with a treatment duration of 1 month or more, and data on efficacy or safety outcomes were available. Reviews, unpublished data, conference papers, notes, non‐RCTs, editorials, letters, case reports, nonhuman studies, and duplicate studies were excluded. Additional exclusion criteria involved studies with overlapping research populations from the same center. One investigator (Yanyan Feng) independently screened titles and abstracts. After removing duplicates, two investigators (Yanyan Feng and Zhaobo Shi) reviewed the full texts of the remaining articles. Any conflicts regarding eligibility were resolved through consensus discussion with a third reviewer (Jiejie Li).

Data from each included trial were collected into predefined Excel spreadsheets. Extracted information included the first author, year of publication, follow‐up time, and number of patients experiencing stroke, MACE, or safety outcomes. We contacted either principal authors or corresponding authors of included RCTs to confirm the number and composite of MACE for this meta‐analysis.

### 2.3 Quality of Evidence Assessment

We utilized the revised risk‐of‐bias 2 tool from the Cochrane Collaboration (Higgins et al. 2011; Sterne et al. 2019) to evaluate the methodological quality of the included RCTs, focusing on the following aspects: randomization process, deviations from intended interventions, missing outcome data, outcome measurement, and selection of reported results. Each domain was categorized as low, some concerns, or high risk of bias by Yanyan Feng and Zhaobo Shi independently. Disagreements were resolved through consensus discussion facilitated by a third reviewer, Jiejie Li.

### 2.4 Statistical Analysis

In this dichotomous meta‐analysis, we synthesized and analyzed data to summarize stroke and MACE events across the included studies, with death as the safety outcome. The RR was used to compare the tongxinluo treatment group with the control group. Heterogeneity between the RCTs was assessed using the *I* ^2^ and *Q* test. When heterogeneity was not significant (*I* ^2^ < 50% or *p* > 0.05), a fixed‐effect model with the inverse variance method was employed to calculate the pooled effect sizes. Otherwise, a random‐effect model with the inverse variance method was used. Subgroup and sensitivity analyses were conducted to identify potential sources of heterogeneity. According to the *Cochrane Handbook for Systematic Reviews of Interventions* (Version 6.5, 2024) (Higgins et al. 2024), when more than 10 studies were included, a funnel plot and Egger test were used to assess small study effects and publication bias, and when publication bias is detected, the trim‐and‐fill method will be used to adjust the results of the pooled effect size. All analyses were conducted using Revman 5.4, STATA 18.0, and R 4.4.2 (R for Statistical Analysis). Statistical significance was set at *p* < 0.05 for all two‐tailed tests.

## 3 Results

### 3.1 Study Inclusion

**FIGURE 1:**
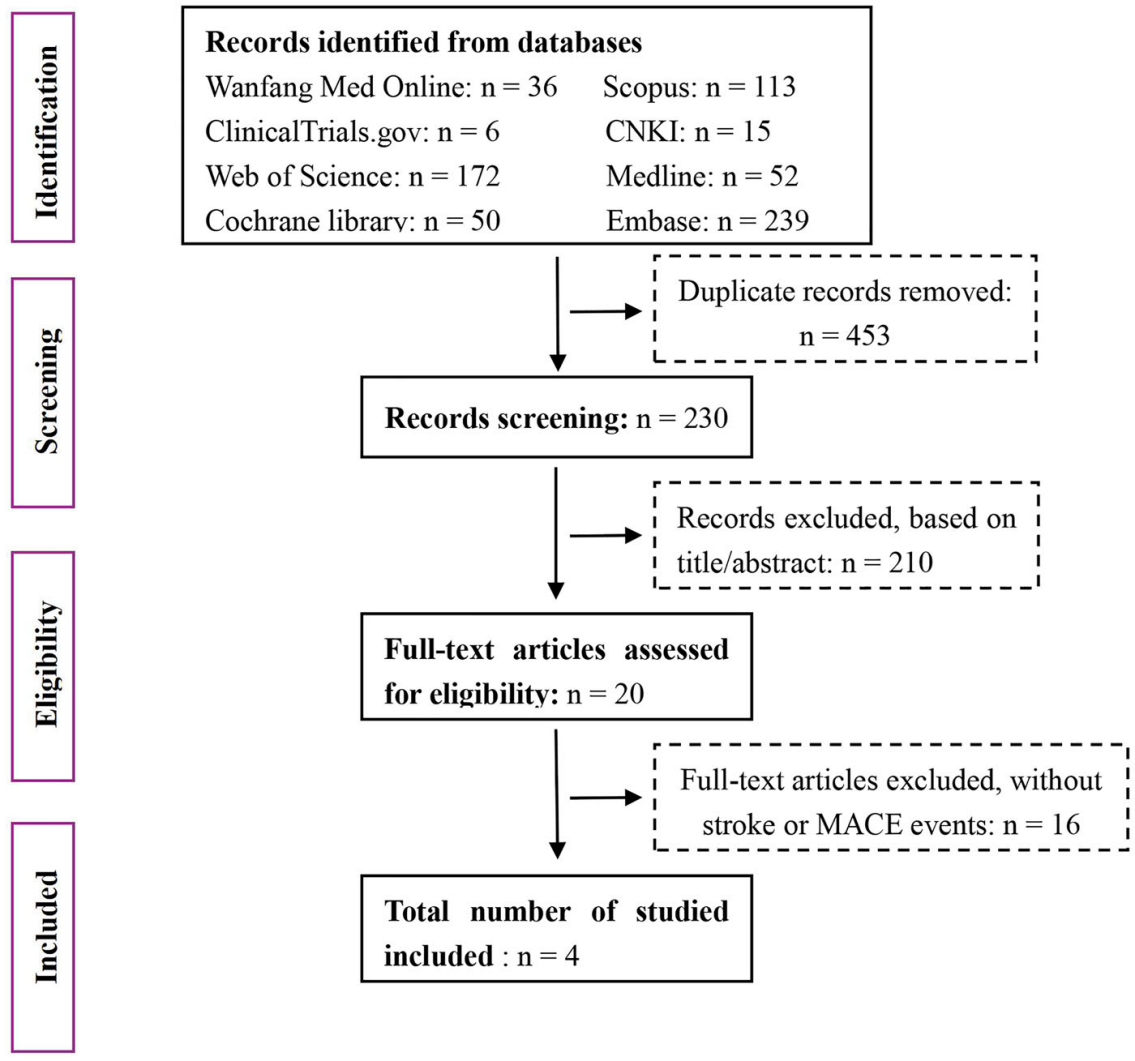
Flow chart of the study selection.

According to the predefined search strategy, we initially retrieved 564 articles from various databases: 36 from Wanfang Med Online, 15 from CNKI, 52 from Medline, 172 from Web of Science, 239 from Embase, 6 from ClinicalTrials.gov, 113 from Scopus, and 50 from the Cochrane Library. After excluding 453 duplicates, 230 articles remained for further screening. Upon reviewing the titles and abstracts, we eliminated 210 articles and then conducted a full‐text review of the remaining 20 articles. Sixteen articles were excluded due to the absence of stroke or MACE events, resulting in four RCTs that met the inclusion criteria (i.e., reporting on stroke or MACE events). The article selection process is illustrated in **Figure** **1**.

### 3.2 Characteristics of Included Studies

Among the four RCTs (Zhang et al. 2019; Zhang et al. 2018; Yang et al. 2023; Dong et al. 2024), a total of 7087 patients were enrolled, with 3545 patients receiving tongxinluo and 3542 receiving placebo. The key characteristics of each study are presented in **Table** **1**. These RCTs focused on ASCVD, specifically acute coronary syndrome, carotid artery plaque, acute ischemic stroke, and acute myocardial infarction. All trials were randomized, placebo‐controlled, and double‐blind. We did not search for other RCTs reporting stroke, MACE, or safety outcomes. In the TISS and CTS‐AMI trials, patients took tongxinluo or placebo three times daily (four capsules each time), with the initial dosage doubled in the TISS trial. In the Lei et al. trial, patients took tongxinluo or placebo three times daily (three capsules each time), while in the carotid artery plaque intervention with tongxinluo capsule (CAPITAL) trial, patients took six capsules twice daily. Follow‐up durations ranged from 1 to 24 months.

**TABLE 1:** Key features of included trials.

Author and Year	Abbreviation	Multicenter	Open label	Follow‐up time (months)	Participants		Dosage (capsule); times/day	Patients
					Tongxinluo	Placebo		
Zhang et al. 2018	NA	Yes	No	1	60	59	3; 3	Acute coronary syndrome
Zhang et al. 2019	CAPITAL	Yes	No	24	607	605	6; 2	Carotid artery plaque
Dong et al. 2024	TISS	Yes	No	3	989	990	4; 3*	Acute ischemic stroke
Yang et al. 2023	CTS‐AMI	Yes	No	12	1889	1888	4; 3	Acute myocardial infarction

* Dosage was double at the first time.

The baseline characteristics of the included RCTs are presented in **Table** **2**. The median age of participants ranged from 8.1 to 61.5 years. The percentage of male participants varied between 58.7% and 77.1%. Body mass index (BMI) ranged from 24.2 to 24.9 kg/m^2^. Blood pressure readings were within the range of 78.8–88.0 mmHg (systolic) and 129.2–146.6 mmHg (diastolic), with the percentage of hypertensive patients ranging from 50.2% to 63.3%. The percentage of patients with diabetes ranged from 18.1% to 28.3%. The proportion of current smokers/smokers varied between 25.4% and 43.8%. In two trials, the percentage of patients with dyslipidemia ranged from 2.8% to 24.0%, and the level of low‐density lipoprotein cholesterol (LDL‐C) ranged from 1.9 to 2.9 mmol/L.

**TABLE 2:** Baseline characteristics of included trials.

Characteristics	Lei et al.		Mei et al.		Yi et al.		Yuejin et al.	
	Tongxinluo *n* = 60	Placebo *n* = 59	Tongxinluo *n* = 607	Placebo *n* = 605	Tongxinluo *n* = 973	Placebo *n* = 973	Tongxinluo *n* = 1889	Placebo *n* = 1888
Age, mean(SD), year	58.7 (10.8)	58.1 (11.6)	61.4 (8.4)	61.4 (8.2)	61.1 (9.7)	60.4 (10.5)	61.4 (12.1)	61.5 (12.1)
Male, No. (%)	39 (65.0)	36 (61.0)	367 (60.5)	355 (58.7)	677 (69.6)	665 (68.3)	1456 (77.1)	1448 (76.7)
BMI, mean (SD), kg/m^2^	24.8 (2.7)	24.4 (2.8)	24.7 (2.9)	24.9 (2.9)	24.2 (3.0)	24.3 (2.7)	24.5 (3.0)	24.5 (3.0)
SBP, mean (SD), mmHg	142.5 (18.6)	140.7 (22.7)	129.4 (11.3)	129.2 (11.4)	148.4 (20.1)	146.6 (20.8)	131.5 (24.6)	132.4 (24.2)
DBP, mean (SD), mmHg	83.1 (12.2)	79.8 (12.4)	78.8 (7.8)	79.0 (8.0)	88.0 (11.9)	88.0 (11.9)	81.9 (16.0)	81.8 (16.0)
Hypertension, No. (%)	38 (63.3)	37 (62.7)	314 (51.7)	342 (56.5)	592 (60.8)	592 (60.8)	948 (50.2)	959 (50.8)
Diabetes, No. (%)	17 (28.3)	17 (28.3)	128 (21.1)	113 (18.7)	252 (25.9)	252 (25.9)	418 (22.1)	398 (21.1)
Dyslipidemia, No. (%)	NA	NA	NA	NA	28 (2.9)	27 (2.8)	454 (24.0)	452 (23.9)
LDL‐C, mean (SD), mmol/L	2.0 (1.2)	1.9 (1.4)	NA	NA	2.8 (0.8)	2.9 (0.9)	NA	NA
Current smoking/smoking, No. (%)	16 (26.7)	15 (25.4)	162 (26.7)	174 (28.8)	378 (38.8)	395 (40.6)	828 (43.8)	790 (41.8)

Abbreviations: BMI, body mass index; SBP, systolic blood pressure; DBP, diastolic blood pressure; LDL‐C: low‐density lipoprotein cholesterol.

### 3.3 Quality of Evidence Assessment

**FIGURE 2:**
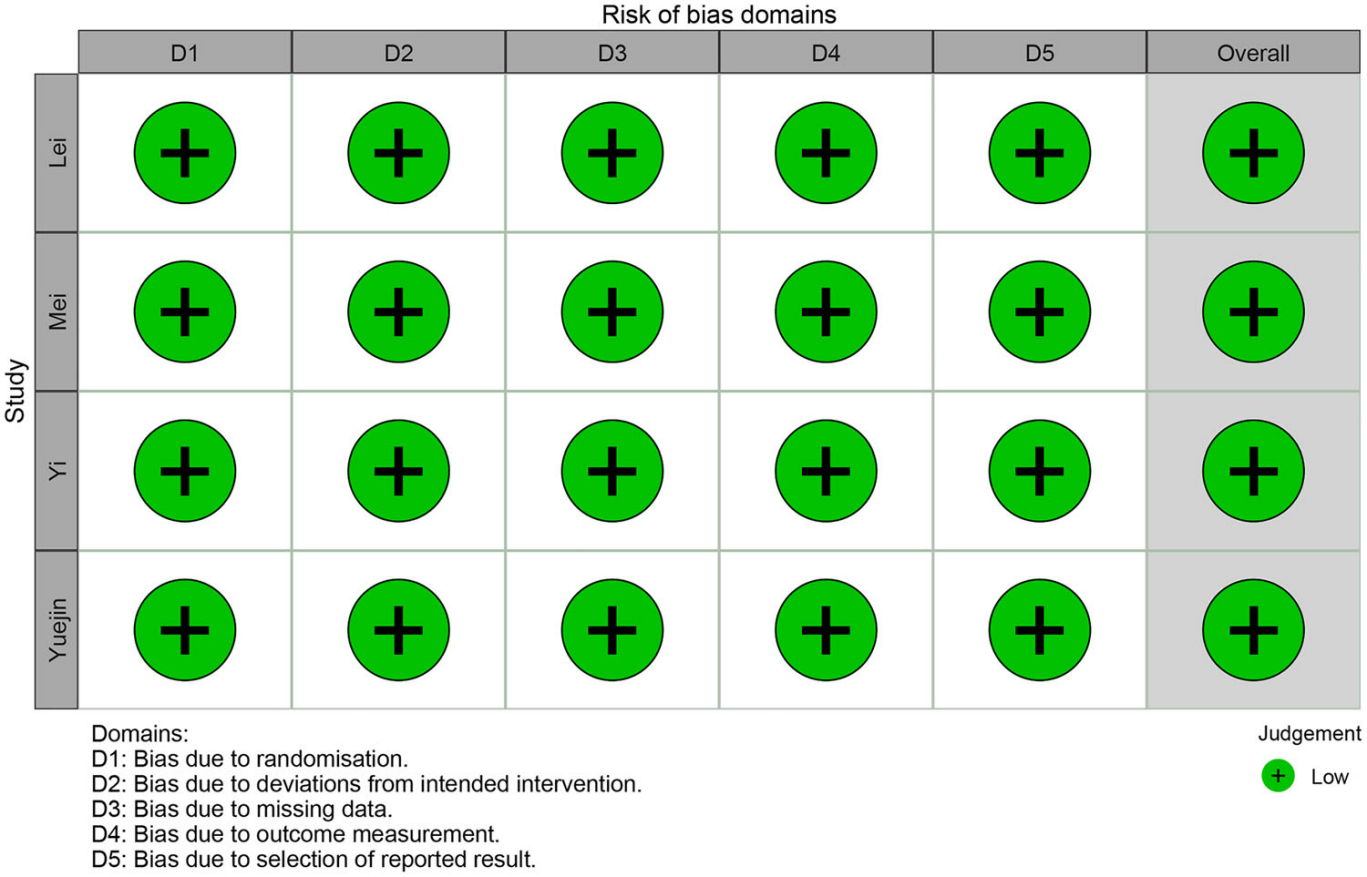
Risk of bias graph for all included trials.

According to the revised Cochrane risk‐of‐bias tool for randomized trials (Higgins et al. 2011; Sterne et al. 2019), all included trials were assessed by investigators. Across all domains, the risk of bias was deemed low. Of the four trials included, allocation concealment was not clearly described in one; the remaining three utilized an automated centralized randomization system. Regarding randomization number generation, sequential randomization numbers were adopted in one study, while the other three employed numbers generated via a computer‐based central randomization system. Consequently, all four trials were classified as having a low risk of bias in the randomization process domain. All trials were double‐blinded, mitigating the risk of deviations from intended interventions and classified as low risk in this domain. Regarding missing data, all articles reported complete data, resulting in a low risk assessment. Although information to fully assess detection bias was incomplete, investigators were blinded to primary outcomes, leading to a low risk classification. The risk of reporting bias was also low, as almost all patient data were analyzed. The summary of risk of bias is presented in **Figure** **2**.

### 3.4 Meta‐Analysis

**FIGURE 3:**
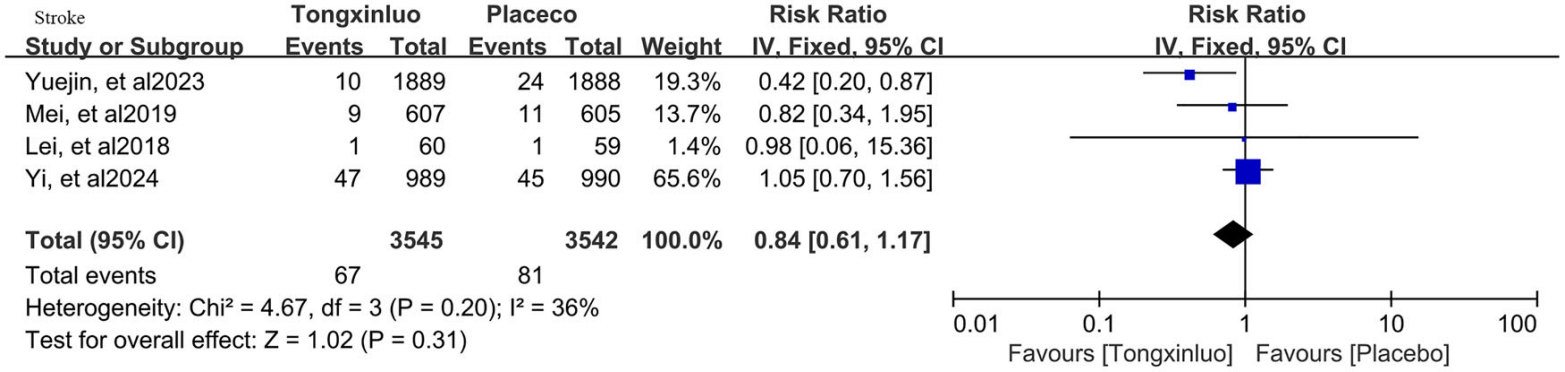
Pooled estimate of tongxinluo's efficacy in preventing stroke.

For the primary outcome of stroke, all four trials reported this event. Among 3545 patients taking tongxinluo, stroke occurred in 67 (1.9%), compared to 81 (2.3%) of 3542 patients taking the placebo. The heterogeneity among trials was low (*p* = 0.20, *I* ^2^ = 36%). Using the fixed‐effect model with the inverse variance method, tongxinluo numerically reduced the risk of stroke in ASCVD patients (RR: 0.84, 95% CI: 0.61–1.17, *p* = 0.31). The results are depicted in **Figure** **3**.

**FIGURE 4:**
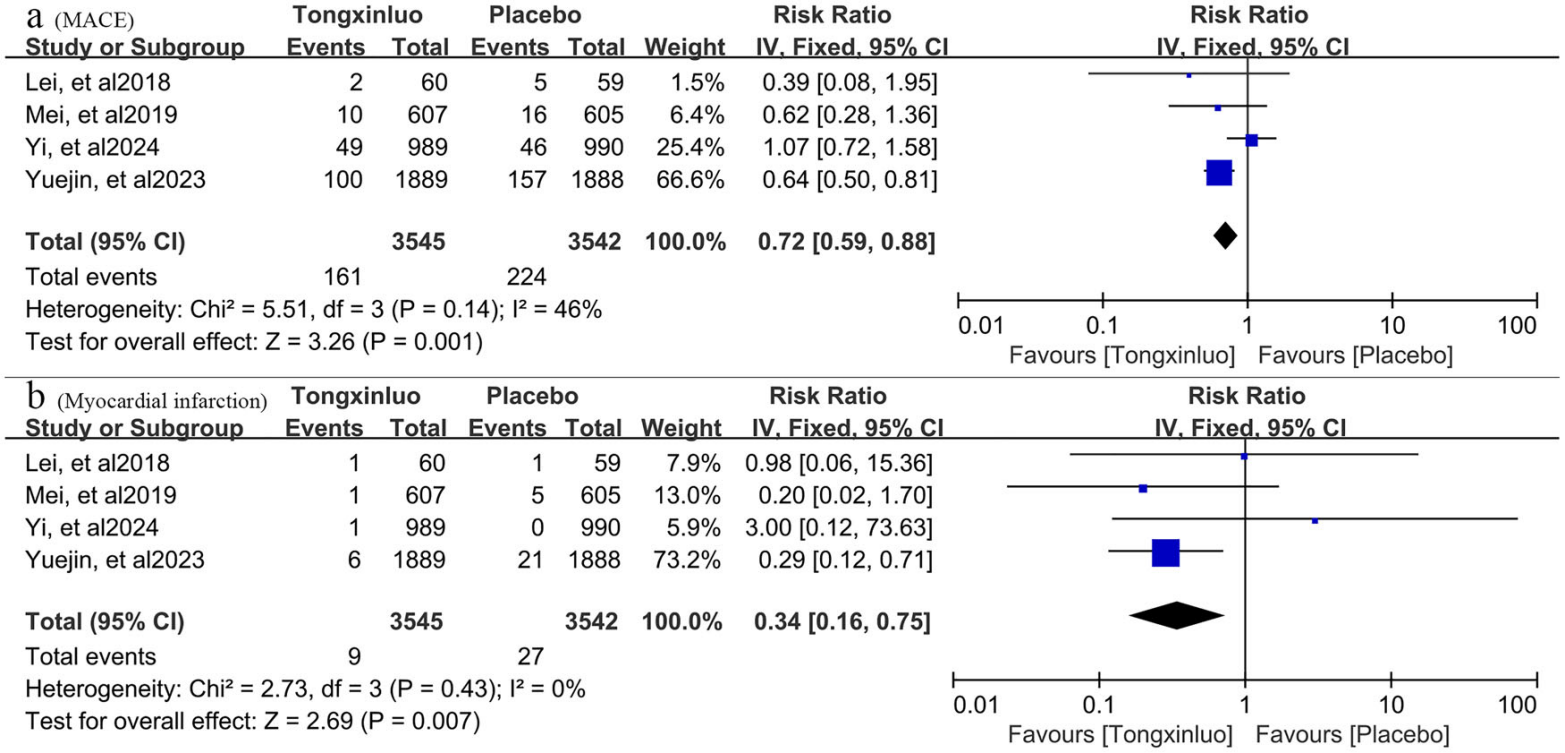
(a) Comprehensive pooled estimate of tongxinluo's efficacy in preventing major adverse cardiovascular events (MACE). (b) Pooled estimate of the preventive effect of tongxinluo against myocardial infarction.

Regarding the outcome of MACE, all four trials reported this event. MACE occurred in 161 (4.5%) of 3545 patients taking tongxinluo and in 224 (6.3%) of 3542 patients taking the placebo. The heterogeneity among trials was low (*p* = 0.14, *I* ^2^ = 46%). Applying the fixed‐effect model with the inverse variance method, tongxinluo reduced the risk of MACE by 28% (RR: 0.72, 95% CI: 0.59–0.88, *p* = 0.001). The results are shown in **Figure** **4** **a**. Participants who received tongxinluo had a RR 0.34 for myocardial infarction (9/3545 vs. 27/3542; 95% CI: 0.16–0.75, *p* = 0.007; **Figure** **4** **b**).

**FIGURE 5:**
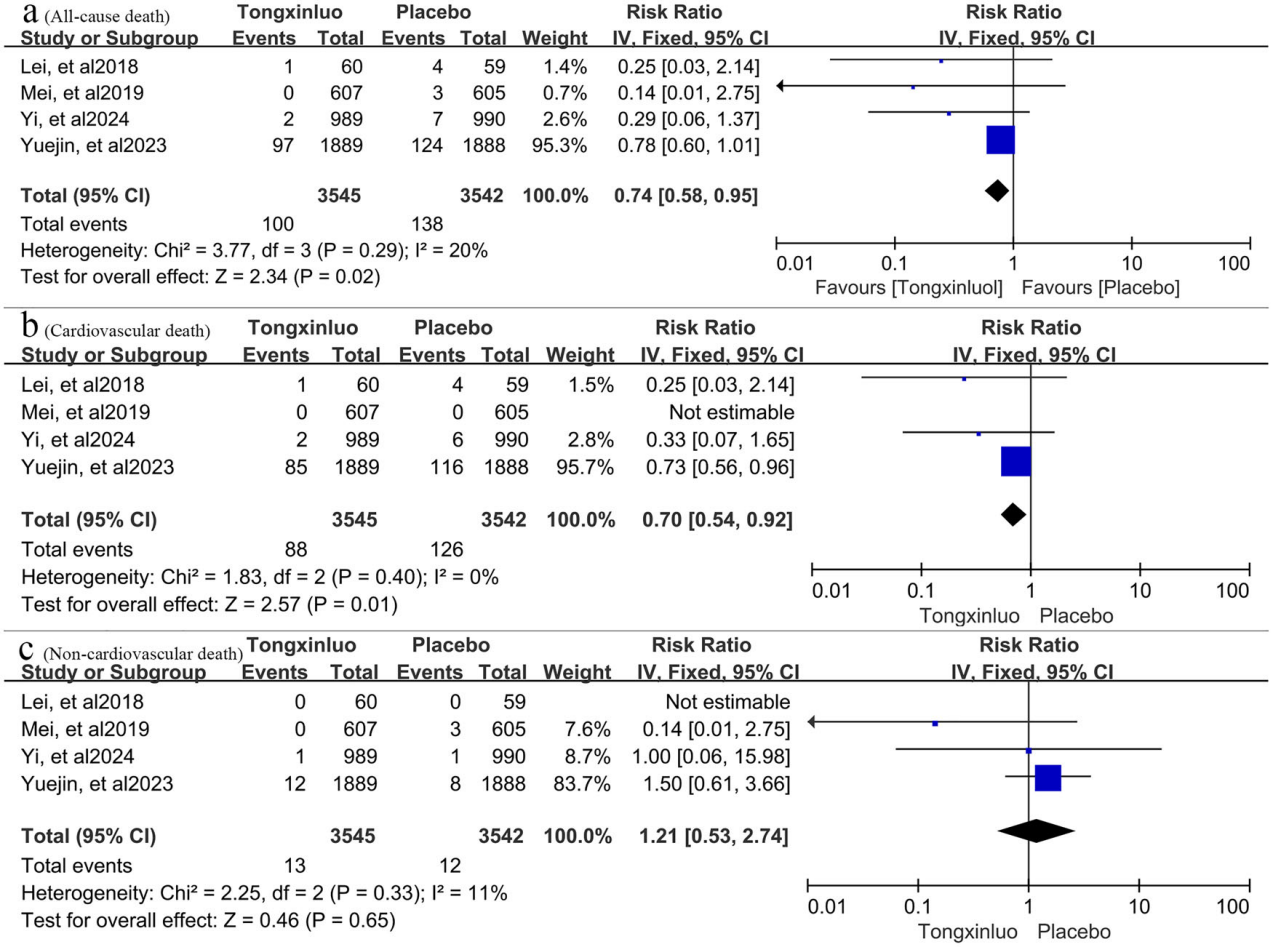
(a) Estimation of the pooled efficacy of tongxinluo in reducing all‐cause mortality. (b) Comprehensive pooled estimation of tongxinluo's efficacy in preventing cardiovascular mortality. (c) Pooled estimation of tongxinluo's efficacy in decreasing non‐cardiovascular mortality.

For the safety outcome, all four trials reported this event. Death occurred in 100 (2.8%) of 3545 patients taking tongxinluo and in 138 (3.9%) of 3542 patients taking the placebo. Death was further classified as all‐cause death, cardiovascular death, and non‐cardiovascular death. The heterogeneity among trials was low (*p* = 0.29, *I* ^2^ = 20%). Using the fixed‐effect model with the inverse variance method, tongxinluo reduced the risk of all‐cause death by 26% (RR 0.74, 95% CI 0.58–0.95, *p* = 0.02) and cardiovascular death by 30% (RR: 0.70, 95% CI: 0.54–0.92, *p* = 0.01) in ASCVD patients compared to placebo. There was no significant difference in non‐cardiovascular death (RR: 1.21, 95% CI: 0.53–2.74, *p* = 0.65). The results are presented in **Figure** **5**.

## 4 Discussion

We conducted a systematic review and analysis of RCTs involving tongxinluo, specifically reporting stroke and MACE events in ASCVD patients. Previous studies have examined tongxinluo in the context of cerebrovascular or cardiovascular disease individually (Li et al. 2021; Lv et al. 2022; Wu et al. 2006; Zhuo et al. 2008). However, our study uniquely evaluated and analyzed the roles of tongxinluo in a comprehensive manner, encompassing acute coronary syndrome, carotid artery plaque, acute ischemic stroke, and acute myocardial infarction. To the best of our knowledge, our study represents the first systematic review and meta‐analysis of tongxinluo specifically in ASCVD patients to date.

Cardio‐cerebrovascular events represent a major global health threat, primarily driven by atherosclerosis. Numerous studies have highlighted the multifaceted involvement of tongxinluo in the progression of atherosclerosis. However, previous research has yielded inconsistent findings regarding tongxinluo's impact on stroke and MACE occurrence. To address this issue, we conducted a systematic review and meta‐analysis of RCTs on tongxinluo, focusing on its effects on stroke and MACE occurrence. Our analysis included four trials. The meta‐analysis showed a nonsignificant reduction in stroke incidence with tongxinluo. Both animal models and patient studies have demonstrated tongxinluo's potent anti‐atherosclerotic effects during plaque formation and development (Zhang et al. 2019; Jiang et al. 2023). With the advancement of research, hsCRP has emerged as a novel risk factor influencing atherosclerotic progression (Bienstock et al. 2024). Two of the included trials (Zhang et al. 2019; Zhang et al. 2018) demonstrated that tongxinluo treatment was associated with a numerical reduction in hsCRP levels. This finding aligns with previous research, suggesting that tongxinluo may exert potent anti‐atherosclerotic effects. However, due to the limited number of eligible trials, we did not perform a meta‐analysis of these data. Furthermore, in animal models, tongxinluo significantly lowers serum lipid levels (Chen et al. 2009; Yuan et al. 2018). Theoretically, tongxinluo may help reduce the occurrence of stroke. More clinical trials were needed to fully explore this query.

Tongxinluo has been found to significantly reduce the incidence of MACCE in patients with acute myocardial infarction, as evidenced in previous studies (Yang et al. 2023). However, in patients with acute ischemic stroke, it does not exert any influence on the occurrence of new recurrent ischemic cerebrovascular disease (Dong et al. 2024). Our meta‐analysis further validates that tongxinluo can markedly decrease the occurrence of MACE, also representing the first evidence of its efficacy in reducing MACE in patients with ASCVD. Consistent with prior research (Yang et al. 2023), tongxinluo decreased the incidence of myocardial infarction among ASCVD patients, which is a component of MACE.

In terms of safety outcomes, all‐cause mortality, cardiovascular mortality, and non‐cardiovascular mortality were assessed, respectively. Individual RCTs indicated that tongxinluo significantly reduced cardiovascular mortality in patients with acute myocardial infarction (Yang et al. 2023). Our meta‐analysis extends this finding to suggest that tongxinluo can decrease both all‐cause and cardiovascular mortality in ASCVD patients. However, tongxinluo was unable to decrease non‐cardiovascular death in patients with ASCVD.

Our review has certain limitations. The number of included trials is limited. In accordance with the *Cochrane Handbook for Systematic Reviews of Interventions* (Higgins et al. 2024), this limited sample size precluded the conduct of the funnel plot analysis and Egger test. Consequently, we were unable to assess publication bias using a powerful and objective approach, which undermines our confidence in the findings. More fundamentally, the small number of studies incorporated in the meta‐analysis also prevents us from performing subgroup analyses. This limitation impairs our ability to determine whether traditional atherosclerosis risk factors, such as age, smoking, dyslipidemia, and hypertension (Herrington et al. 2016), exert an impact on the efficacy of tongxinluo in preventing stroke and MACE.

Additionally, the included studies adopted different dosing regimens for tongxinluo. Although the statistical heterogeneity among these studies was low, and the dosages used were generally consistent with the package insert, clinical heterogeneity persisted. This clinical variability may compromise the reliability and generalizability of our conclusions. Furthermore, some studies failed to report baseline LDL‐C levels, which hinder a comprehensive evaluation of the background lipid‐lowering therapy and its potential interaction with tongxinluo.

Of the included trials, four incorporated a placebo control group. Among these four, three explicitly stated that no other traditional Chinese patent medicines were used during the study period. However, the remaining trial did not specify whether concurrent use of other traditional Chinese medicines was permitted. This ambiguity may have led to confounding due to mixed intervention measures, thereby introducing uncertainty into our conclusions.

Lastly, due to insufficient data and the limited number of eligible studies in the meta‐analysis, we are unable to utilize meta‐regression. This makes it impossible for us to assess whether the baseline or clinical characteristics of the included patients influenced our results. However, we conducted a sensitivity analysis by removing each included paper individually, and our results remained unchanged. Notably, the heterogeneity of the included trials was low, which enhances the reliability of our conclusions.

Based on our meta‐analysis results, tongxinluo has been primarily investigated in atherosclerosis diseases. PAD, resulting from atherosclerotic occlusive disease, affects approximately 10% of individuals under 70 and 20% of individuals over 70, predominantly those in their 60s and 70s (Ouriel 2001; Golledge 2022; Hirsch et al. 2001; Song et al. 2019). Despite thorough database and clinical trial searches, no clinical studies on tongxinluo for PAD were found, resulting in incomplete conclusions. Future studies focusing on tongxinluo for PAD are anticipated. Additionally, while searching for RCTs, we identified high‐quality trials that were excluded due to nonreporting of stroke or MACE events (Zhang et al. 2010), further reducing the number of included studies. Research on tongxinluo has mainly targeted coronary heart diseases, with limited focus on stroke events. Future scholars should conduct more comprehensive RCTs, particularly for cerebrovascular diseases.

## 5 Conclusions

In summary, tongxinluo has shown a tendency to decrease the incidence of stroke, as well as lower the incidence of MACE and mortality in ASCVD patients, supporting its use in clinical practice for atherosclerosis diseases.

## Author Contributions

**Yanyan Feng**: writing – original draft, writing – review and editing, formal analysis, data curation, software, resources, methodology, project administration, visualization. **Zhaobo Shi**: methodology, conceptualization, data curation, formal analysis, validation. **Jiewen Zhang**: formal analysis, data curation, investigation, supervision. **Jiejie Li**: data curation, formal analysis, supervision, methodology, conceptualization, validation.

## Funding

The authors have nothing to report.

## Supporting information




**Supporting Information**: brb371232‐sup‐0001‐SuppMat.docx

## Data Availability

The datasets generated during the current study are available from the corresponding and first authors on reasonable request.
