# Supplementary material for: Tongxinluo for the Secondary Prevention of Atherosclerotic Disease: A Systematic Review and Meta‐Analysis of Randomized Clinical Trials
Source: Brain Behav. 2026 Mar 12;16(3):e71232. doi: 10.1002/brb3.71232 (PMC13093790; doi:10.1002/brb3.71232)
Supplement: Supplementary file 1 — Supporting Information: brb371232‐sup‐0001‐SuppMat.docx [file BRB3-16-e71232-s001.docx]

**20/11/2024**

**Wanfang Med Online: 36 results.**

1. 通心络

资源类型：(中文期刊 OR 外文期刊)

1. ((((随机对照试验) OR 对照临床试验随机) OR 临床试验随机) OR 随机对照临床试验) OR 随机临床试验

资源类型：(中文期刊 OR 外文期刊)

1. 动脉粥样硬化

资源类型：(中文期刊 OR 外文期刊)

1. (((脑卒中) OR 脑血管意外) OR 脑血管中风) OR 脑中风

资源类型：(中文期刊 OR 外文期刊)

1. (心肌梗死) OR 心肌梗塞

资源类型：(中文期刊 OR 外文期刊)

6. ((脑梗死) OR 脑梗塞) OR 脑血栓

资源类型：(中文期刊 OR 外文期刊)

7. ((((脑出血) OR 颅内出血) OR 脑内出血) OR 脑实质出血) OR 蛛网膜下腔出血

资源类型：(中文期刊 OR 外文期刊)

8. (外周动脉疾病) OR 外周血管疾病

资源类型：(中文期刊 OR 外文期刊)

9. 心肌血管重建

资源类型：(中文期刊 OR 外文期刊)

10. #1 AND #2 AND #3

11. #1 AND #2 AND #4

12. #1 AND #2 AND #5

13. #1 AND #2 AND #6

14. #1 AND #2 AND #7

15. #1 AND #2 AND #8

16. #1 AND #2 AND #9

**China National Knowledge Infrastructure (CNKI): 15 results.**

1.（题名/关键词/摘要：通心络）资源范围：期刊;

2.（题名/关键词/摘要：随机对照试验）OR（题名/关键词/摘要：对照临床试验随机(精确)）OR（题名/关键词/摘要：临床试验随机(精确)）OR（题名/关键词/摘要：随机对照临床试验(精确)）OR（题名/关键词/摘要：随机临床试验(精确)）资源范围：期刊;

3.（题名/关键词/摘要：动脉粥样硬化） 资源范围：期刊

4.（主要主题：心肌梗塞(本)）OR（题名/关键词/摘要：心肌梗塞(精确)）资源范围：期刊;

5.（主要主题：脑梗塞(本)）OR（题名/关键词/摘要：脑梗塞(精确)）OR（题名/关键词/摘要：脑血栓(精确)） 资源范围：期刊;

6.（主要主题：卒中(本)）OR（题名/关键词/摘要：脑血管意外(精确)）OR（题名/关键词/摘要：脑血管中风(精确)）OR（题名/关键词/摘要：脑中风(精确)）资源范围：期刊;

7.（主要主题：脑出血(本)）OR（题名/关键词/摘要：颅内出血(精确)）OR（题名/关键词/摘要：脑内出血(精确)）OR（题名/关键词/摘要：脑实质出血(精确)）OR（题名/关键词/摘要：蛛网膜下腔出血(精确)）资源范围：期刊;

8.（主要主题：外周动脉疾病(本)）OR（题名/关键词/摘要：外周血管疾病(精确)）资源范围：期刊;

9.（题名/关键词/摘要：心肌血管重建） 资源范围：期刊;

10. #1 AND #2 AND #3

11. #1 AND #2 AND #4

12. #1 AND #2 AND #5

13. #1 AND #2 AND #6

14. #1 AND #2 AND #7

15. #1 AND #2 AND #8

16. #1 AND #2 AND #9

**Medline: 52 results.**

1 "tongxinluo"[Supplementary Concept] OR "tongxinluo"[All Fields] OR (((xin, tong[Author] OR tong, xin[Author]) OR tong xin[Author] OR tong xin[Investigator]) AND "luo"[All Fields])

2 "randomized controlled trial"[Publication Type] OR "randomized controlled trials as topic"[MeSH Terms] OR "randomized controlled trial"[All Fields] OR "randomised controlled trial"[All Fields] OR "randomized controlled trial"[Publication Type] OR "randomized controlled trials as topic"[MeSH Terms] OR "randomized clinical trial"[All Fields] OR "randomised clinical trial"[All Fields] OR "controlled clinical trial"[Publication Type] OR "controlled clinical trials as topic"[MeSH Terms] OR "controlled clinical trial"[All Fields] OR "clinical trial"[Publication Type] OR "clinical trials as topic"[MeSH Terms] OR "clinical trial"[All Fields] OR "clinical trial"[Publication Type] OR "clinical trials as topic"[MeSH Terms] OR "clinical trial"[All Fields]

3 "atherosclerosis"[MeSH Terms] OR "atherosclerosis"[All Fields] OR "atheroscleroses"[All Fields] OR "atherosclerosis"[MeSH Terms] OR "atherosclerosis"[All Fields] OR "atheroscleroses"[All Fields] OR "atherosclerosis"[MeSH Terms] OR "atherosclerosis"[All Fields] OR "atherogenesis"[All Fields] OR "atherosclerosis"[MeSH Terms] OR "atherosclerosis"[All Fields] OR "atherogenesis"[All Fields]

4 "stroke"[MeSH Terms] OR "stroke"[All Fields] OR "strokes"[All Fields] OR "stroke s"[All Fields] OR ("stroke"[MeSH Terms] OR "stroke"[All Fields] OR ("cerebrovascular"[All Fields] AND "accident"[All Fields]) OR "cerebrovascular accident"[All Fields]) OR ("stroke"[MeSH Terms] OR "stroke"[All Fields] OR ("brain"[All Fields] AND "vascular"[All Fields] AND "accident"[All Fields]) OR "brain vascular accident"[All Fields]) OR ("apoplexies"[All Fields] OR "stroke"[MeSH Terms] OR "stroke"[All Fields] OR "apoplexy"[All Fields]) OR ("stroke"[MeSH Terms] OR "stroke"[All Fields] OR "cva"[All Fields])

5 "brain infarction"[MeSH Terms] OR ("brain"[All Fields] AND "infarction"[All Fields]) OR "brain infarction"[All Fields] OR ("brain infarction"[MeSH Terms] OR ("brain"[All Fields] AND "infarction"[All Fields]) OR "brain infarction"[All Fields] OR ("brain"[All Fields] AND "infarct"[All Fields]) OR "brain infarct"[All Fields]) OR ("cerebral infarction"[MeSH Terms] OR ("cerebral"[All Fields] AND "infarction"[All Fields]) OR "cerebral infarction"[All Fields])

6 "cerebral haemorrhage"[All Fields] OR "cerebral hemorrhage"[MeSH Terms] OR ("cerebral"[All Fields] AND "hemorrhage"[All Fields]) OR "cerebral hemorrhage"[All Fields] OR ("intracerebral haemorrhage"[All Fields] OR "cerebral hemorrhage"[MeSH Terms] OR ("cerebral"[All Fields] AND "hemorrhage"[All Fields]) OR "cerebral hemorrhage"[All Fields] OR ("intracerebral"[All Fields] AND "hemorrhage"[All Fields]) OR "intracerebral hemorrhage"[All Fields]) OR ("cerebral hemorrhage"[MeSH Terms] OR ("cerebral"[All Fields] AND "hemorrhage"[All Fields]) OR "cerebral hemorrhage"[All Fields] OR ("cerebrum"[All Fields] AND "hemorrhage"[All Fields])) OR ("brain haemorrhage"[All Fields] OR "intracranial hemorrhages"[MeSH Terms] OR ("intracranial"[All Fields] AND "hemorrhages"[All Fields]) OR "intracranial hemorrhages"[All Fields] OR ("brain"[All Fields] AND "hemorrhage"[All Fields]) OR "brain hemorrhage"[All Fields]) OR ("subarachnoid haemorrhage"[All Fields] OR "subarachnoid hemorrhage"[MeSH Terms] OR ("subarachnoid"[All Fields] AND "hemorrhage"[All Fields]) OR "subarachnoid hemorrhage"[All Fields]) OR "SAH"[All Fields]

7 "peripheral arterial disease"[MeSH Terms] OR ("peripheral"[All Fields] AND "arterial"[All Fields] AND "disease"[All Fields]) OR "peripheral arterial disease"[All Fields] OR ("peripheral arterial disease"[MeSH Terms] OR ("peripheral"[All Fields] AND "arterial"[All Fields] AND "disease"[All Fields]) OR "peripheral arterial disease"[All Fields] OR ("peripheral"[All Fields] AND "artery"[All Fields] AND "diseases"[All Fields]) OR "peripheral artery diseases"[All Fields])

8 "myocardial infarction"[MeSH Terms] OR ("myocardial"[All Fields] AND "infarction"[All Fields]) OR "myocardial infarction"[All Fields] OR ("myocardial infarction"[MeSH Terms] OR ("myocardial"[All Fields] AND "infarction"[All Fields]) OR "myocardial infarction"[All Fields] OR ("heart"[All Fields] AND "attack"[All Fields]) OR "heart attack"[All Fields]) OR ("myocardial infarction"[MeSH Terms] OR ("myocardial"[All Fields] AND "infarction"[All Fields]) OR "myocardial infarction"[All Fields] OR ("myocardial"[All Fields] AND "infarct"[All Fields]) OR "myocardial infarct"[All Fields]) OR ("myocardial infarction"[MeSH Terms] OR ("myocardial"[All Fields] AND "infarction"[All Fields]) OR "myocardial infarction"[All Fields] OR ("cardiovascular"[All Fields] AND "stroke"[All Fields]) OR "cardiovascular stroke"[All Fields])

9 (("coronaries"[All Fields] OR "heart"[MeSH Terms] OR "heart"[All Fields] OR "coronary"[All Fields]) AND ("revascularisation"[All Fields] OR "revascularisations"[All Fields] OR "revascularise"[All Fields] OR "revascularised"[All Fields] OR "revascularising"[All Fields] OR "revascularization"[All Fields] OR "revascularizations"[All Fields] OR "revascularize"[All Fields] OR "revascularized"[All Fields] OR "revascularizes"[All Fields] OR "revascularizing"[All Fields])) OR ("percutaneous coronary intervention"[MeSH Terms] OR ("percutaneous"[All Fields] AND "coronary"[All Fields] AND "intervention"[All Fields]) OR "percutaneous coronary intervention"[All Fields])

10. #1 AND #2 AND #3

11. #1 AND #2 AND #4

12. #1 AND #2 AND #5

13. #1 AND #2 AND #6

14. #1 AND #2 AND #7

15. #1 AND #2 AND #8

16. #1 AND #2 AND #9

**Web of Science: 172 results.**

1. "(TS=(tongxinluo)) OR TS=(tong xin luo) and Preprint Citation Index (Exclude–Database)"
2. "((((TS=(randomized controlled trial)) OR TS=( randomized clinical trial)) OR TS=(controlled clinical trial)) OR TS=(clinical trial)) OR TS=(clinical study) "
3. "(((TS=(atherosclerosis)) OR TS=(atheroscleroses)) OR TS=(atherogenesis)) OR TS=(atherogenesis)"
4. "((((TS=(stroke)) OR TS=(cerebrovascular accident)) OR TS=(brain vascular accident)) OR TS=(apoplexy)) OR TS=(CVA) "
5. "((TS=(brain infarction)) OR TS=(brain infarct)) OR TS=(cerebral infarction) "
6. "(((((TS=(cerebral hemorrhage)) OR TS=(intracerebral hemorrhage)) OR TS=(cerebrum hemorrhage)) OR TS=(brain hemorrhage)) OR TS=(subarachnoid hemorrhage)) OR TS=(SAH ) "
7. "(TS=(peripheral arterial disease)) OR TS=(peripheral artery diseases) "
8. "(((TS=(myocardial infarction)) OR TS=(heart attack)) OR TS=(myocardial infarct)) OR TS=(cardiovascular stroke) "
9. "(TS=(coronary revascularization)) OR TS=(percutaneous coronary intervention) "

10. #1 AND #2 AND #3

11. #1 AND #2 AND #4

12. #1 AND #2 AND #5

13. #1 AND #2 AND #6

14. #1 AND #2 AND #7

15. #1 AND #2 AND #8

16. #1 AND #2 AND #9

**Cochrane library: 50 results**

1. (tongxinluo):ti,ab,kw OR (tong xin luo):ti,ab,kw (Word variations have been searched)

2. (randomized controlled trial):ti,ab,kw OR (randomized clinical trial):ti,ab,kw OR (controlled clinical trial):ti,ab,kw OR (clinical trial):ti,ab,kw OR (clinical study):ti,ab,kw (Word variations have been searched)

3. (atherosclerosis):ti,ab,kw OR (atheroscleroses):ti,ab,kw OR (atherogenesis):ti,ab,kw OR (atherogenesis):ti,ab,kw (Word variations have been searched)

4. (stroke):ti,ab,kw OR (cerebrovascular accident):ti,ab,kw OR (brain vascular accident):ti,ab,kw OR (apoplexy):ti,ab,kw OR (CVA):ti,ab,kw (Word variations have been searched)

5. (brain infarction):ti,ab,kw OR (brain infarct):ti,ab,kw OR (cerebral infarction):ti,ab,kw (Word variations have been searched)

6. (cerebral hemorrhage):ti,ab,kw OR (intracerebral hemorrhage):ti,ab,kw OR (cerebrum hemorrhage):ti,ab,kw OR (brain hemorrhage):ti,ab,kw OR (subarachnoid hemorrhage):ti,ab,kw (Word variations have been searched)

7. (peripheral arterial disease):ti,ab,kw OR (peripheral artery diseases):ti,ab,kw (Word variations have been searched)

8. (myocardial infarction):ti,ab,kw OR (heart attack):ti,ab,kw OR (myocardial infarct):ti,ab,kw OR (cardiovascular stroke):ti,ab,kw (Word variations have been searched)

9. (coronary revascularization):ti,ab,kw OR (percutaneous coronary intervention):ti,ab,kw (Word variations have been searched)

10. #1 AND #2 AND #3

11. #1 AND #2 AND #4

12. #1 AND #2 AND #5

13. #1 AND #2 AND #6

14. #1 AND #2 AND #7

15. #1 AND #2 AND #8

16. #1 AND #2 AND #9

**Embase: 239 results**

1. tongxinluo OR (tong AND xin AND luo)
2. randomized AND controlled AND trial OR (controlled AND clinical AND trial) OR (clinical AND trial) OR (clinical AND study) OR (randomized AND clinical AND trial)
3. atherosclerosis OR atheroscleroses OR atherogenesis
4. stroke OR (cerebrovascular AND accident) OR (brain AND vascular AND accident) OR apoplexy OR cva
5. brain AND infarction OR (brain AND infarct) OR (cerebral AND infarction)
6. cerebral AND hemorrhage OR (intracerebral AND hemorrhage) OR (brain AND hemorrhage) OR (subarachnoid AND hemorrhage) OR sah OR (cerebrum AND hemorrhage)
7. myocardial AND infarction OR (heart AND attack) OR (myocardial AND infarct) OR (cardiovascular AND stroke)
8. peripheral AND arterial AND disease OR (peripheral AND artery AND diseases)
9. coronary AND revascularization OR (percutaneous AND coronary AND intervention)
10. #1 AND #2 AND #3
11. #1 AND #2 AND #4
12. #1 AND #2 AND #5
13. #1 AND #2 AND #6
14. #1 AND #2 AND #7
15. #1 AND #2 AND #8
16. #1 AND #2 AND #9

**ClinicalTrials.gov: 6 results**

Tongxinluo

**Scopus: 113 results**

1. tongxinluo OR (tong AND xin AND luo)
2. randomized AND controlled AND trial OR (controlled AND clinical AND trial) OR (clinical AND trial) OR (clinical AND study) OR (randomized AND clinical AND trial)
3. atherosclerosis OR atheroscleroses OR atherogenesis
4. stroke OR (cerebrovascular AND accident) OR (brain AND vascular AND accident) OR apoplexy OR cva
5. brain AND infarction OR (brain AND infarct) OR (cerebral AND infarction)
6. cerebral AND hemorrhage OR (intracerebral AND hemorrhage) OR (brain AND hemorrhage) OR (subarachnoid AND hemorrhage) OR sah OR (cerebrum AND hemorrhage)
7. myocardial AND infarction OR (heart AND attack) OR (myocardial AND infarct) OR (cardiovascular AND stroke)
8. peripheral AND arterial AND disease OR (peripheral AND artery AND diseases)
9. coronary AND revascularization OR (percutaneous AND coronary AND intervention)
10. #1 AND #2 AND #3
11. #1 AND #2 AND #4
12. #1 AND #2 AND #5
13. #1 AND #2 AND #6
14. #1 AND #2 AND #7
15. #1 AND #2 AND #8
16. #1 AND #2 AND #9
